# Beyond the tumor: endocrine and metabolic dysregulation in intracranial germ cell tumors – a retrospective cohort study

**DOI:** 10.1055/s-0045-1812300

**Published:** 2025-10-27

**Authors:** Mayco José Reinaldi Serra, Angela Maria Spinola-Castro, Nasjla Saba da Silva, Andrea Cappellano, Paola Matiko Martins Okuda, Adriana Aparecida Siviero-Miachon

**Affiliations:** 1 Universidade Federal de São Paulo, São Paulo SP, Brazil.; 2 Universidade Lusiada, Santos SP, Brazil.; 3 Grupo de Apoio ao Adolescente e à Criança com Câncer, São Paulo SP, Brazil.

**Keywords:** Germinoma, Central Nervous System Diseases, Hypopituitarism, Obesity, Diabetes Insipidus, Radiotherapy

## Abstract

**Background:**

Intracranial germ cell tumors (iGCTs) often lead to endocrine-metabolic complications; however, their long-term effects are not well understood and characterized.

**Objective:**

To evaluate endocrine-metabolic dysfunction before and after iGCTs treatment.

**Methods:**

The present retrospective study included 99 patients with iGCTs treated at a tertiary hospital. Endocrine and metabolic parameters were assessed before and after treatment.

**Results:**

A male sex predominance was observed (81.8%). The leading site was pineal (44.4%), and 67.7% of the tumors were classified as germinoma. Radiotherapy was performed in 82.8% of the cases (58.5% cranial and 41.5% craniospinal). At diagnosis, the incidences of gonadotropin-independent precocious puberty and diabetes insipidus were 15.2% and 48.2%, respectively. Significant endocrine-metabolic changes in patients with iGCTs were observed after treatment, as 72.3% of patients required hormone replacement, 60% had growth hormone deficiency, and dyslipidemia was observed in 49.2% of patients. Overweight increased from 24.2 to 35.4% after treatment, while obesity increased from 10.1 to 15.4%. There was an increase in growth hormone deficiency, hypothyroidism, and hypogonadism, while prolactin levels significantly decreased after treatment. An older age at diagnosis was associated with a lower risk of hypocortisolism ( *p* = 0.005). Regarding sex, females had lower height Z-scores and a higher frequency of growth hormone deficiency compared with males. Tumor-related mortality was reported in 22.2% of patients, occurring on average 2.2 years postdiagnosis.

**Conclusion:**

The high prevalence of endocrine-metabolic complications following radiotherapy highlights the necessity of ongoing monitoring. The treatment demonstrated significant efficacy, as reflected by the notable survival rates. Early intervention is crucial for improving the long-term quality of life.

## INTRODUCTION

Intracranial germ cell tumors (iGCTs) primarily affect children and young adults, accounting for ∼ 3.3% of cases in children aged 0 to 14 years. 1 The observed incidence is 5.5% in children with central nervous system (CNS) neoplasms undergoing radiotherapy (RT) and up to 14% in cases of CNS neoplasms in Asian countries. 2 3 Germinomas are the most prevalent iGCTs in the pineal and sellar regions, and the primary tumor markers are usually β-human chorionic gonadotropin (β-hCG) and α-fetoprotein (AFP). Furthermore, bifocal iGCTs involving both areas may co-occur, with an incidence ranging from 2 to 41%. 4 5

Various reports have shown that pineal tumors typically present with symptoms of intracranial hypertension, along with other manifestations, such as altered levels of consciousness, convulsions, psychiatric symptoms, and ataxia. Hypothalamic-pituitary (HP) hormonal deficiencies are rare at diagnosis but may be underestimated. Tumors producing β-hCG are notable for causing gonadotropin-independent precocious puberty (GIPP), predominantly in male children. 5 6 7 Conversely, sellar-located iGCTs may show varying degrees of multiple hormonal deficiencies at diagnosis, with diabetes insipidus (DI) being a key manifestation. Diabetes insipidus may be present months or years before a neoplasm is diagnosed and is essential for differentiating it from other sellar tumors, such as craniopharyngioma, in which DI is rare at presentation. 5 6 7 8

Few studies have evaluated endocrine and metabolic dysfunctions in survivors of iGCTs. Nonetheless, it is a critical issue to be addressed, as multiple HP deficiencies may exist, and patients may benefit from early interventions. It is worth noting that patients frequently search for medical evaluation with endocrine symptoms, which may represent the main manifestations of these tumors. Furthermore, being aware of the significance of late effects after treatment and their frequency may increase the likelihood of survival. Still, it undoubtedly leads to a better quality of life for all affected individuals. Thus, the present study aimed to describe the endocrine-metabolic manifestations in children and young adults with iGCTs, as well as their clinical and auxological characteristics at diagnosis and after treatment.

## METHODS

### Study population

The study population consisted of 99 children, adolescents, and young adults aged 0 to 39 years diagnosed with iGCTs and followed up at the hospital of Grupo de Apoio ao Adolescente e à Criança com Câncer (GRAACC) between 1992 and 2020. The Ethics and Research Committee of Universidade Federal de São Paulo and the hospital of GRAACC approved the present historical cohort study.

### Procedures

The diagnosis of iGCTs involved a comprehensive assessment comprising clinical evaluation, laboratory analyses, quantification of βhCG and AFP in both serum and cerebrospinal fluid (CSF), and cranio-spinal magnetic resonance imaging (MRI). The specific treatment protocols implemented both before and after 2013 have been detailed in prior studies. 9 10 Patients' tumors were characterized as germinomas (G-iGCT) if they presented with negative AFP levels (< 5 ng/dL) and βhCG levels below 50 IU/L (prior to 2013) or 200 IU/L (after 2013). In cases of uncertain diagnosis, confirmation was obtained via biopsy. Both established protocols consistently involved initial chemotherapy (CT) followed by RT. The spatial extent of the RT was determined by various criteria, notably by the response to CT and the presence of residual tumor markers.

The following parameters were evaluated in patients with iGCTs:

- Clinical characteristics: age, sex, tumor location, histological type (G-iGCTs, or non-germinomas - NG-iGCTs), presence of CNS metastases at diagnosis, serum and CSF tumor markers β-hCG and AFP, employment of CT, and/or RT, dose and site, if tumor resection, and autologous hematopoietic stem cell transplantation (HSCT);
- Anthropometric assessment: Height and body mass index (BMI) were converted into Z-scores (Z BMI) at diagnosis and posttherapy in the appraisal closest to 2020. 11 12 Overweight was defined as a Z BMI of > 1.0 standard deviation (SD), obesity as a Z BMI of > 2.0 SD, and severe obesity as a Z BMI of > 3.0 SD. Short stature was defined as a height Z-score (Z height) < -2.0 SD. Final height was considered when growth velocity was < 2 cm/year and bone age > 14 years in girls and > 16 years in boys. Anthropometric variables were compared after using recombinant human growth hormone (rhGH);
- Endocrine-metabolic assessment: Hormone deficiencies, assessed at diagnosis and posttherapy, including:
  ○ Diabetes insipidus: Serum sodium above 145 mEq/L, low urinary density (lower than 1,005 in urinalysis), and urine output greater than 4 mL/kg/h;
  ○ Growth hormone (GH) deficiency: Short stature, Z height < 1.0 SD below target height, growth velocity < 25th percentile for age and sex, negative GH stimulation tests (< 5 ng/mL), and/or low insulin-like growth factor 1 (IGF-1);
  ○ Hyperprolactinemia: Prolactin above 20 ng/mL;
  ○ Hypothyroidism: Free T4 (FT4) and thyroid-stimulating hormone (TSH) levels were evaluated using chemiluminescence immunoassay. Central hypothyroidism was considered when FT4 was low and TSH was normal or low, whereas primary hypothyroidism was considered when TSH was elevated and FT4 was normal or low;
  ○ Hypocortisolism: Basal morning cortisol evaluated by chemiluminescence immunoassay lower than 3 μg/dL, and/or less than 18 μg/dL after insulin tolerance test;
  ○ GIPP: Elevated serum β-hCG and total testosterone in boys;
○ Dyslipidemia (DLP): Low-density lipoprotein cholesterol (LDL-c) > 130 mg/dL, high-density lipoprotein cholesterol (HDL-c) < 40 mg/dL, and/or elevated triglyceride levels (TG > 100 mg/dL in children up to 10 years or > 130 mg/dL in those older than 10 years); 13
○ Insulin resistance: Elevated homeostasis model assessment of insulin resistance 1-insulin resistance (HOMA1-IR), calculated by the formula: fasting plasma glucose (mg/dl) × 0.0555 × fasting plasma insulin (μIU/ml) divided by 22.5. Homeostasis model assessment of insulin resistance 1-insulin resistance cut-offs for children up to 10 years were based on the 95th percentile by sex and age, while 3.16 and 2.35 were considered for adolescents and young adults, respectively. 14 15 16 17

### Statistical analysis

Before the statistical analysis, missing data were imputed using the expectation-maximization (EM) method to estimate missing values based on existing data, thereby reducing bias and improving robustness. 18 19 The paired Student's *t* -test was used to assess differences in clinical, anthropometric, and endocrine-metabolic characteristics before and after treatment. A one-way analysis of variance (ANOVA) for repeated measures was used to compare pre- and post-treatment endocrine-metabolic assessments and their variations according to age, sex, tumor histology (G-iGCTs and NG-iGCTs), RT employment and site, and follow-up comorbidities. Binary logistic regression was constructed with forward likelihood-ratio selection (forward likelihood ratio method): variables were added step-by-step, each kept only if it significantly lowered the model's -2 log-likelihood ( *p* < 0.05); the procedure stopped when no new variable improved the fit, leaving only the predictors that truly contributed information. 20 21 This procedure enabled the evaluation of the relationship between clinical characteristics and endocrine-metabolic outcomes, examining the influence of variables such as age, sex, anthropometric data, tumor markers, CNS metastases, histology, tumor location, endocrinological features, treatment type, tumor recurrence, and death on endocrine and metabolic outcomes. The results are expressed as odds ratios (ORs) with 95%CIs. Statistical analyses were conducted using IBM SPSS Statistics for Windows (IBM Corp.) software, version 23.0, with a significance level of 0.05.

## RESULTS

### Study population

The study encompassed 99 patients, with a mean age of 12.7 (range: 4.8–25.2) years at diagnosis, and predominantly male ( *n* = 80/99; 81.8%), with a sex ratio of 4.2:1.

Tumors were most frequently identified in the pineal gland ( *n* = 44/99; 44%). Pineal iGCTs predominantly occurred in males (53.7%), whereas tumors in the sellar region were more prevalent among females (73.7%). Positive serum and CSF β-hCG markers were detected in 34/99 (34.3%) and 36/99 (36.4%) patients, respectively. Concurrent positive serum and CSF AFP markers were found in 20/99 (20.2%) patients. The primary histological classification was G-iGCTs ( *n* = 67/99; 67.7%), with metastases in 13/99 (13.1%).

Treatment was initiated at the hospital of GRAACC in 87/99 patients (87.8%). With regards to therapy, 97/99 patients (97.9%) underwent CT, and 82/99 (82.8%) received RT, as following: cranial in 48/82 (58.5%), and craniospinal in 34/82 (41.5%). Tumor resection was performed in 31/99 subjects (31.3%), and autologous HSCT in 9/99 (9.1%). Tumor-related mortality was reported in 22/99 (22.2%), occurring on average 2.2 years postdiagnosis. After treatment, 12/99 patients (12.2%) failed to maintain their follow-up. Consequently, 65/99 patients (65.5%) were available for posttreatment endocrine-metabolic analysis. The last posttreatment evaluation occurred ∼ 90.6 months (SD = 64.0) after diagnosis. The clinical characteristics of the study population at the time of diagnosis are presented in Table 1 .

**Table 1:** Clinical characteristics of patients with intracranial germ cell tumors at diagnosis

Variable	n (%)
Male sex	81 (81.8)
Positive serum and CSF β-hCG	34 and 36 (34.3 and 36.4)
Positive serum and CSF AFP	20 and 20 (20.2 and 20.2)
Histology	G-iGCT 67 (67.7)
	NG-iGCT 32 (32.3)
Location of tumor	Pineal: 44 (44.4)
	Bifocal: 28 (28.3)
	Sellar: 25 (25.3)
	Basal ganglia: 1 (1)
	Thalamus: 1 (1)
Presence of metastases	13 (13.1)
Chemotherapy use	97 (97.9)
Radiotherapy use and site	Cranial: 48 (58.5)
	Craniospinal: 34 (41.5)
	Total: 82 (82.8)
	Dose above 30Gy: 96.3
Tumor resection	31 (31.3)
Autologous HSCT	9 (9.1)

Abbreviations: CSF, cerebrospinal fluid; β-hCG, human chorionic gonadotropin; AFP, α-fetoprotein; G-iGCTs, germinoma intracranial germ cell tumors; NG-iGCTs, non-germinomatous intracranial germ cell tumors; HSCT, hematopoietic stem cell transplantation.

### Anthropometric characteristics

A non-significant increase in Z BMI was observed, rising from -0.20 to 0.31 (t (64) = −1.474; *p* = 0.145). This was accompanied by a discernible, albeit non-significant shift toward higher rates of overweight and obesity among patients posttreatment. Specifically, the proportion of patients classified as overweight increased from 24.2 to 35.4%, and those with obesity increased from 10.1 to 15.4% ( *p* > 0.05).

**Figure 1:**
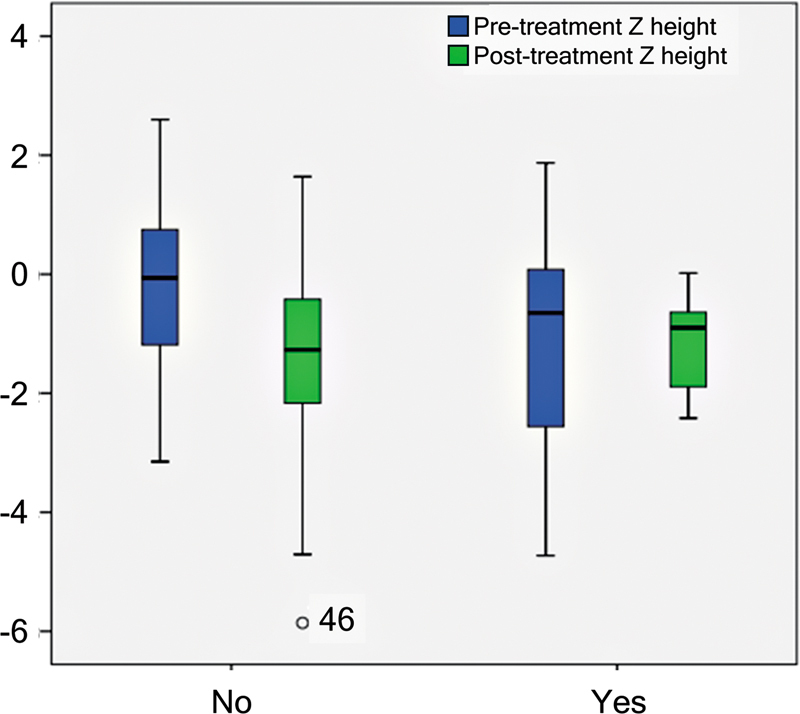
Pre- and posttreatment height Z-scores (Z height) of patients according to recombinant human growth hormone (rhGH) use. Notes: Patients receiving rhGH experienced less height loss than non-rhGH patients after treatment; however, this difference was not statistically significant (t (1) = 0.048; *p* = 0.827).

Anthropometric analysis revealed a significant decrease in Z height from -0.31 at diagnosis to -1.36 posttreatment (t (64) = 4.890; *p* ≤ 0.001). The prevalence of short stature increased from 11.1 to 16.9%. Among the 39/65 patients with GH deficiency, 13/39 (33.3%) received rhGH. Patients receiving rhGH experienced less height loss than non-rhGH patients after treatment; however, this difference was not statistically significant (t (1) = 0.048; *p* = 0.827) ( Figure 1 ).

### Endocrine and metabolic characteristics

Endocrine abnormalities were prevalent at diagnosis and posttreatment ( Table 2 ). The study revealed significant endocrinological and metabolic changes in patients with iGCTs following treatment, as 72.3% of patients required hormone replacement, 15.2% had GIPP, and 48.2% had DI at diagnosis. Persistent endocrine issues and additional metabolic complications post-treatment, such as insulin resistance (72.3%) and DLP (49.2%) were also prevalent. Other posttreatment comorbidities included epilepsy (26.2%) and thyroid nodule monitoring (3.1%).

**Table 2:** Incidence of endocrinological dysfunction pre- ( *n* = 99) and post-treatment ( *n* = 65) in patients with intracranial germ cell tumors

Endocrinopathy		Pretreatment		Posttreatment		*P* -value
		N (99)*	%	N (65)*	%	
**Diabetes insipidus**		48	48.5	34	52.3	> 0.05
**Hypothyroidism**	Central	42	42.4	25	38.5	> 0.05
	Primary	−	**-**	9	13.8	
**Growth hormone deficiency**		45	45.5	39	60	> 0.05
**Hyperprolactinemia**		73	73.7	28	43.1	< 0.05
**Hypogonadism**		25	25.3	23	35.4	> 0.05
**Precocious puberty**		15	15.2	−	−	
**Hypocortisolism**		48	48.5	28	43.1	> 0.05

Notes: The number of posttreatment participants decreased from 99 to 65 due to the loss of patients because of death and/or loss of follow-up. Pretreatment precocious puberty is independent of gonadotropin levels.

Several endocrine disorders increased after treatment, although not all statistically significant. The prevalence of DI, GH deficiency, hypogonadism, and hypothyroidism has increased. Conversely, hyperprolactinemia decreased significantly ( Table 2 ).

Age at diagnosis significantly influenced clinical, oncological, and endocrine-metabolic characteristics. Younger patients were more likely to have NG-iGCTs (OR = 0.880; 95%CI = 0.783 -0.989; *p* = 0.032). Older patients had an 18.4% higher chance of metastases per year of age (OR = 1.184; 95%CI = 1.025–1.367, *p* = 0.021) and a 14.5% lower chance of developing hypocortisolism (OR = 0.855; 95%CI = 0.766–0.955; *p* = 0.005).

Regarding sex differences, females presented with lower Z height and higher rates of endocrine disorders. Tumor location impacted clinical presentation, with sellar and bifocal tumors associated with higher rates of endocrine disorders pre- and posttreatment. Patients with NG-iGCTs exhibited higher serum and CSF AFP levels, with the serum AFP level increasing the probability of NG-iGCTs by 5.87 times (β = 1.77; 95%CI = 2.04–16.82; *p* = 0.001). This group also exhibited lower posttreatment Z-height and Z-BMI values. Germinomas patients had higher pretreatment hyperprolactinemia and posttreatment DLP. Craniospinal RT was associated with increased endocrine and metabolic complications, highlighting the potential long-term effects of this treatment. Notably, mortality rates showed a significant sex disparity, with males experiencing a higher mortality rate than females (26.2% versus 5.3%; *p* = 0.049) ( Table 3 ).

**Table 3:** Effects of sex, histology, location, and radiotherapy employment pre- and posttreatment on clinical, oncological, and endocrine-metabolic characteristics of iGCTs patients †

		Variable	F (df)	*p* -value
**Sex** **(male versus female)**	**Pretreatment**	Height Z-scores	34.24 (1.63)	≤ 0.001
		Diabetes insipidus	11.58 (1.63)	0.001
		Hypocortisolism	25.50 (1.63)	≤ 0.001
		Hypothyroidism	7.95 (1.63)	0.006
		Growth hormone deficiency	23.18 (1.63)	< 0.001
	**Posttreatment**	Diabetes insipidus	10.42 (1.63)	0.002
		Hypocortisolism	19.08 (1.63)	≤ 0.001
		Hypothyroidism	6.869	0.011
**Location of tumor (pineal, bifocal, sellar, basal ganglia, thalamus)**	**Pretreatment**	Height z-scores (sellar versus all)*	3.40 (1.63)	0.023
		Diabetes insipidus (bifocal versus all)*	16.77 (1.63)	≤ 0.001
		Hypocortisolism (sellar versus all)*	6.68 (1.63)	0.001
		Growth hormone deficiency (sellar versus all)*	2.83 (1.63)	0.046
	**Posttreatment**	Diabetes insipidus (bifocal versus all)*	12.36 (1.63)	≤ 0.001
		Hypocortisolism (sellar versus all)*	11.94 (1.63)	≤ 0.001
		Hypothyroidism (bifocal versus all)*	5.61 (1.63)	0.002
		Elevated triglycerides (bifocal versus all)*	2.72 (1.63)	0.052
**Histology (G-iGCTs versus NG-iGCTs)**	**Pretreatment**	Hyperprolactinemia	9.90 (1.63)	0.003
	**Posttreatment**	Growth hormone deficiency	4.20 (1.63)	0.045
		Dyslipidemia	7.34 (1.63)	0.009
		Elevated triglycerides	4.70 (1.63)	0.034
**Radiotherapy site (not performed, cranial or craniospinal)**	**Posttreatment**	Hyperprolactinemia (craniospinal and cranial versus not)*	6.41 (1.63)	0.003
		Growth hormone deficiency (craniospinal versus all)*	4.62 (1.63)	0.013
		Epilepsy (craniospinal versus all)*	5.73 (1.63)	0.005
		Elevated low-density lipoprotein cholesterol (craniospinal versus all)*	4.54 (1.63)	0.015

Abbreviations: df, degrees of freedom; F, F-statistic; G-iGCTs, germinomas; iGCTs, intracranial germ cell tumors; NG-iGCT, non-germinomas.

Notes: F = ratio of the mean square between groups to the mean square within groups (ANOVA F-statistic). Values marked with *differ significantly in the Least Significant Difference (LSD) post-hoc test at *p* ≤ 0.05.

† Only variables that present significant differences in the pre- and posttreatment are present in Table 3.

Endocrine changes observed in iGCT patients were linked to metabolic complications. Patients with overweight or obesity and insulin resistance showed higher rates of endocrine and metabolic dysfunction posttreatment ( Table 4 ).

**Table 4:** Endocrine and metabolic changes after treatment in intracranial germ cell tumors according to body mass index and insulin resistance ^†^

Endocrinopathy/metabolic change		F (df)	*p* -value
**Obesity or overweight**	Hypogonadism	10.14 (1.63)	0.002
	Insulin resistance	6.90 (1.63)	0.011
	Dyslipidemia	9.70 (1.63)	0.003
	Decreased HDL-c	12.95 (1.63)	0.001
	Elevated triglycerides	12.35 (1.63)	0.001
**Insulin resistance**	Hypocortisolism	4.59 (1.63)	0.036
	Hypothyroidism	5.65 (1.63)	0.020
	Dyslipidemia	4.78 (1.63)	0.033
	Decreased HDL-c	4.70 (1.63)	0.034

Abbreviations: df, degrees of freedom; F, F-statistic; HDL-c, high-density lipoprotein cholesterol.

Note: ^†^ Only variables that present significant differences in the posttreatment are present in Table 4.

## DISCUSSION

The present study examined the endocrine-metabolic and anthropometric traits of patients with iGCTs, focusing on the disease's impact and its treatment.

The study cohort comprised 99 patients at diagnosis and 65 patients after treatment. This is due to mortality and loss of follow-up (22 and 12 patients, respectively). Although the hospital of GRAACC is a national reference in pediatric oncology, follow-up after treatment is highly challenging in a country as large as Brazil.

The median age at diagnosis was 12.1 years, consistent with previous studies whose predominance was in adolescence. 1 4 7 Younger patients were more likely to have NG-iGCTs and hypocortisolism. However, Partenope et al. did not observe an association between age and any specific hypothalamic pituitary (HP) dysfunction before or after treatment. 22

Male sex predominance in iGCTs (4.2:1) was slightly higher than in other studies (2.5–3:1). 23 24 25 26 27 Pineal tumors were most frequently observed in males (53.7%), and sellar tumors among females (73.7%). This observation highlights the relationship between female patients and multiple HP dysfunctions, as pineal tumors have been reported to have a lower risk of hormone deficiencies. 22 23 24 Additionally, a higher mortality rate was observed in males. This marginal statistical finding should be analyzed in the light of a complex and multifaceted relationship between sex and prognosis in patients with iGCTs. In this cohort, a pineal site predominance was noted in males, which may be related to acute neurologic complications and a higher mortality attributable to delayed diagnosis or shortcomings in therapeutic access in a resource-limited country.

The presence of metastases was observed in 13.1% of patients, which aligns with rates reported in other studies, such as 10.9% by Partenope et al. and 17.3% by Kurucu et al. 22 26 This finding emphasizes the critical role of neuroimaging during diagnosis, especially considering the frequent occurrence of metastases in the early stages of iGCT. 4

At diagnosis, the incidence of excess weight (overweight or obesity) was 24.2%, which increased to 35.4% posttreatment. While considering obesity, the incidence rose from 10.1 at diagnosis to 15.4% after treatment. Wang et al. reported an incidence of 46.2% of excess weight and 13.2% of obesity after iGCT treatment, with a median follow-up of 27 months (2.2 years). Their study also noted a trend toward an elevated Z BMI within the first 6 years posttreatment. 28 Similarly, Partenope et al. observed an increase in obesity from pre- to posttreatment (5.5–14.5%), with a follow-up period of 78.9 months (6.6 years). 22 Excess weight at diagnosis and posttreatment was linked to hypogonadism, but not to other HP isolated deficiencies. Furthermore, excess weight was also linked to high LDL-c, low HDL-c, and hypertriglyceridemia, suggesting an increased prevalence of metabolic syndrome components among iGCT survivors.

Z height showed a decrease after treatment, given that 58.5% of patients received cranial RT at doses greater than 30 Gy. The administration of rhGH may have mitigated the reduction in height. Growth hormone deficiency occurred posttreatment in 60% of patients, mainly female sex, and sellar iGCTs. 22 29 Nonetheless, among patients with GH deficiency, only 33.3% received rhGH therapy. This may be explained by several factors, including the minimum interval free of disease to consider rhGH use, as well as discussions among endocrinologists, oncologists, and families to weigh the benefits and risks of rhGH use in this group of patients.

The reported incidence of HP axis deficiencies at diagnosis varies in the literature. In the present study, the most frequently observed HP axis deficiency was DI and hypocortisolism (both 48.5%), followed by GH deficiency (45.5%). These findings are consistent with other studies. 24 25

Gonadotropin-independent precocious puberty is a crucial diagnostic tool in identifying iGCTs, once β-hCG is a marker for some NG-iGCT. 4 Gonadotropin-independent precocious puberty was observed exclusively in males in this study (15.2%), showing a slightly higher incidence compared with other investigations. For instance, García et al. reported an incidence of 8.3%, Kurucu et al. reported 7.7%, and Odagiri et al reported 13.6%. 26 30 31 Hyperprolactinemia at diagnosis suggests damage to the pituitary stalk and reduced dopaminergic inhibition. A posttreatment decrease in prolactin levels may be attributed to the effects of cranial RT, correlating with panhypopituitarism. 23 24 32 Diabetes insipidus was present in 48.2% of patients at diagnosis and increased post-treatment, but not significantly. As GIPP, this endocrine dysfunction is also a key symptom in the differential diagnosis of HP lesions, with iGCTs being the most prevalent. 22 24 29

Despite a slight elevation in the incidence of most HP axis deficiencies after treatment, this change did not reach statistical significance. Hormone replacement therapy (HRT) is typically required in most cases. In the present study, 72.3% of cases needed HRT posttreatment, which is consistent with other authors, such as Chang et al. (61.5%), Partenope et al. (67.3%), and Sawamura et al. (68%). 22 25 33 However, some studies have reported HRT being required in more than 90% of patients following treatment, which is dependent on tumor location and RT dosage. 25 30 34

The prevalence of DLP was lower than reported in the literature, characterized by increased LDL-c and triglycerides, and decreased HDL-c. 28 Other factors, such as histology, tumor location, and craniospinal RT may have influenced DLP. Nonetheless, this study observed that insulin resistance, like weight gain, increased the risk of DLP and reduced HDL-c. Insulin resistance should be evaluated with caution, due to the inherent difficulty in establishing an optimal HOMA1-IR cut-off value for defining insulin resistance in children and adolescents. The heterogeneity of aging in this cohort is remarkable, and the interpretation of HOMA1-IR values during puberty differs considerably from those during adulthood. 14 15 16 17 Moreover, it is essential to emphasize that the substantially higher prevalence of elevated HOMA1-IR underscores the importance of metabolic evaluation in iGCT survivors, even in non-obese patients.

Limitations of this study include the problems associated with retrospective studies, such as the inclusion of patients from various geographical locations and treatment protocols, which may result in heterogeneity of the sample. To address the challenges posed by missing data, the EM algorithm was utilized, thereby reducing bias and enhancing robustness. Anthropometric measurements were consistently conducted using standardized equipment; however, they were not recorded by a single observer. Endocrine-metabolic assessments were not routinely performed at diagnosis, resulting in incomplete data for some patients.

In conclusion, the present study reveals a high prevalence of endocrine-metabolic dysfunctions in iGCT patients, both at diagnosis and posttreatment. Diabetes insipidus and GIPP are significant at diagnosis, showing a high risk of complications posttreatment, stressing the need for thorough monitoring and timely intervention. Craniospinal RT is associated with increased GH deficiency and metabolic issues, underscoring the importance of personalized treatment and extended follow-up. Future research should focus on mitigating the endocrine effects of RT and enhancing metabolic outcomes for individuals with iGCT survivors.
